# Protocol for the evaluation of a decision aid for women with a breech-presenting baby [ISRCTN14570598]

**DOI:** 10.1186/1471-2393-4-26

**Published:** 2004-12-20

**Authors:** Christine L Roberts, Natasha Nassar, Alexandra Barratt, Camille H Raynes-Greenow, Brian Peat, David Henderson-Smart

**Affiliations:** 1 Centre for Perinatal Health Services Research, QEII Building DO2, University of Sydney, NSW 2006, Australia; 2 School of Public Health, Edward Ford Building, University of Sydney NSW 2006, Australia; 3 Women's and Children's Hospital, Dept. of Perinatal Medicine, 1st Floor, Queen Victoria Building, Brougham Place, North Adelaide, SA 5006, Australia

## Abstract

**Background:**

There is now good evidence about the management options for pregnant women with a breech presentation (buttocks or feet rather than head-first) at term; external cephalic version (ECV) – the turning of a breech baby to a head-down position and/or planned caesarean section (CS). Each of these options has benefits and risks and the relative importance of these vary for each woman, subject to her personal values and preferences, a situation where a decision aid may be helpful.

Decision aids are designed to assist patients and their doctors in making informed decisions using information that is unbiased and based on high quality research evidence. Decision aids are non-directive in the sense that they do not aim to steer the user towards any one option, but rather to support decision making which is informed and consistent with personal values.

The ECV decision aid was developed using the Ottawa Decision Support Framework, including a systematic review of the evidence about the benefits and risks of the options for breech pregnancy. It comprises an audiotape with a supplementary booklet and worksheet, a format that can be taken home and discussed with a partner. This project aims to evaluate the ECV decision aid for women with a breech presenting baby in late pregnancy.

**Study design:**

We aim to evaluate the effectiveness of the decision aid compared with usual care in a randomised controlled trial in maternity hospitals that offer ECV. The study group will receive the decision aid in addition to usual care and the control group will receive standard information on management options for breech presentation from their usual pregnancy care provider. Approximately 184 women with a single breech-presenting baby at greater than 34 weeks gestation and who are clinically eligible for ECV will be recruited for the trial.

The primary outcomes of the study are knowledge, decisional conflict, anxiety and satisfaction with decision-making that will be assessed using self-administered questionnaires. The decision aid is not intended to influence either the uptake of either ECV or planned CS, however we will monitor health service utilisation rates and maternal and perinatal outcomes.

## Background

### Breech presentation

Breech presentation occurs when a baby presents with the buttocks or feet rather than head first (cephalic presentation). As breech presentation is related to both fetal size and gestational age, the incidence decreases as pregnancy progresses to 3–4% by full-term[1,2]. Decades of controversy over the safe management of breech birth at term has recently been resolved by an international multicentre randomised controlled trial (the Term Breech Trial, TBT) of planned vaginal breech birth versus planned caesarean section (CS)[3]. This trial was stopped prematurely because of overwhelming benefit favouring planned CS, with a relative risk of 0.33 (95%CI 0.19–0.56) for perinatal/neonatal mortality or serious neonatal morbidity[3]. The TBT results were subsequently added to two small trials in a Cochrane Systematic Review[4]. The reduction in perinatal morbidity and mortality was even more pronounced when the analyses were limited to births in low perinatal mortality countries, such as Australia (RR 0.13; 95%CI 0.05–0.31)[4]. However, planned caesarean section was associated with increased maternal morbidity (RR 1.29; 95% CI 1.03–1.61)[4].

These results have dramatically altered a woman's options if she has a breech presentation at term, as CS is now offered as the safest and in many institutions the only, management option. This change has occurred rapidly: The TBT was published in October 2000 and the rate of vaginal breech birth in NSW declined from 17% in 1999 to 14% in 2000 and 4.5% in 2001[5]. However, while safer than vaginal breech birth, planned CS is not without risk[6,7]. Complications include increased risk of pulmonary embolism, infection [8-10], bleeding[9,11], damage to bladder and bowel[12], slower recovery from the birth[12,13], longer hospitalisation[11], respiratory difficulties for the baby [14-16], delayed bonding and breastfeeding[17,18], and compromise of future obstetric performance[17,19-21]. Therefore, the best way to avoid the increased risks associated with term breech presentation is to avoid it altogether, and this is possible via external cephalic version.

### External cephalic version (ECV) of the breech-presenting baby

External cephalic version (ECV) is the turning of a breech baby to a cephalic presentation. Systematic review of six well designed randomised controlled trials demonstrates that among women with breech presentation in late pregnancy, ECV reduces both breech presentations in labour (RR = 0.42, 95%CI 0.35–0.50) and caesarean sections (RR = 0.52. 95%CI 0.39–0.71)[22].

Despite clear evidence of effectiveness and potential benefit, many women decline ECV for a variety of reasons. Both breech presentation and ECV success rates are strongly influenced by parity, with success rates reported as low as 25% for women having their first baby[23]. For other women, the inconvenience of extra clinic visits and the need for an IV line for tocolysis may be deterrents[24,25]. Approximately 35% of women undergoing ECV report mild or moderate discomfort during the procedure[26]. Other complications of ECV are either uncommon (e.g. transient fetal bradycardia [12%] or dizziness and palpitations from tocolysis [4%]) or rare (<1%) (e.g. profound fetal bradycardia, preterm labour, premature rupture of membranes and bleeding)[26]. The remote possibility of emergency CS (e.g. because of placental abruption following the procedure) is also recognised. For these and other reasons, women may have a preference for planned CS. An Australian study of decision making for CS conducted in 1996 included 62 women with a breech presentation[27]. Of these, 39 women were offered ECV and 12 (31%) "decided against it". Further, 37 women were offered vaginal breech birth but 14 (38%) women chose CS[27].

### Women's views and information needs

To obtain data on Australian women's views and information needs about ECV we undertook a cross-sectional study of women's knowledge, attitudes and decision-making preferences for the management of breech presentation[28]. Of 174 pregnant women respondents (97% response rate), almost 90% preferred vaginal delivery but only 66% had heard of ECV. After a brief written explanation of ECV 39% would choose ECV, 22% were unsure and 39% would not choose ECV. The reasons for not choosing ECV included concerns about safety for the baby (13%), that ECV doesn't guarantee vaginal delivery (12%) and preference for a caesarean section anyway (8%). Importantly, 95% of pregnant women wanted involvement in decision-making about breech presentation.

### Patient participation in clinical decision making

It is now recognised that many consumers want to participate in clinical decisions about their health [29-31]. NHMRC states that good medical decision making should take account of patients' preferences and values[29], thus challenging health professionals to find ways of involving consumers/patients in decisions about their health. Yet little is currently known about how this can be effectively achieved. One method is to provide information to consumers about treatment options and likely outcomes. To assist informed decision making, such information must be unbiased and based on current, high quality, quantitative research evidence. However, patient information materials are often outdated, inaccurate, omit relevant data, fail to give a balanced view and ignore uncertainties and scientific controversies[31,32].

To help patients take a more active role in important clinical decisions, decision aids based on latest research evidence are being developed by several centres (for example the Ottawa Health Decision Center in Canada and the Foundation for Informed Medical Decision Making in the USA). Decision aids are defined by the Cochrane Collaboration[33] as "interventions designed to help people make specific and deliberative choices among options by providing (at minimum) information on the options and outcomes relevant to the person's health status". Additional strategies may include providing: information on the disease/condition; the probabilities of outcomes tailored to a person's health risk factors; an explicit values clarification exercise; examples of others' decisions; and guidance and coaching in the steps of decision making[33]. Decision aids are *non-directive *in the sense that they do not aim to steer the user towards any one option, but rather to support decision making which is informed, consistent with personal values and acted upon[34]. Decision aids have been found to improve patient knowledge and create more realistic expectations, to reduce decisional conflict (uncertainty about the course of action) and to stimulate patients to be more active in decision making without increasing anxiety[35].

Currently only 38 decision aids worldwide have been developed and carefully evaluated in randomised controlled trials[33,35]. Examples include hormone replacement therapy for postmenopausal women, anticoagulants for atrial fibrillation, PSA testing for prostate cancer and prenatal genetic screening. Until the publication of a series of evidence-based leaflets in the United Kingdom in 2002[36,37], no decision aids have been developed and evaluated in the context of obstetric care, although this is an area in which consumers are known to want to participate actively in decision making[38]. An Australian survey of 790 postpartum women found not having an active say in decisions about pregnancy care was associated with a sixfold increase in dissatisfaction among primiparas and a fifteen fold increase among multiparas[38]. Similarly in the UK, postpartum women rated an explanation of procedures and involvement in decision making as most important to satisfaction with care[39]. Further, neither obstetricians nor midwives appreciated the importance to women of "being told the major risks for each procedure"[39].

### Decision making and breech presentation

The management of breech presentation is a clinical decision that fulfils Eddy's criteria for a decision in which patients' values and preferences should be included[40]. The outcomes for the breech management options (ECV and planned CS), and women's preferences for the relative value of benefits compared to risks are variable and could result in decisional conflict. For such a clinical decision, a decision aid would be expected to improve patient knowledge and create more realistic expectations, to reduce decisional conflict and to stimulate patients to be more active in decision making without increasing anxiety[35].

### Development and pilot-testing of the decision aid

In 2002 we developed an evidence-based decision aid for women with a breech presentation in late pregnancy. In developing the decision aid we utilised the NHMRC guideline "How to prepare and present information for consumers of health services"[41] (developed in 1999 by a team led by Dr Barratt), and the Ottawa framework established and rigorously tested by the Ottawa Health Decision Center[34]. The decision aid includes a Workbook, Audiotape/CD and Worksheet. The workbook highlights key points (similar to a slide presentation) and the audiotape/CD connects these points in a narrative format, providing more detail than the workbook. The worksheet is a one-page sheet to be completed by the woman to record her decision making steps, to list any questions she needs answered before deciding, and to indicate her preferred role in this decision (she should decide, her health care provider should decide, they should decide together). Most importantly, the DA was designed to be non-directive in that it did not aim to steer the user towards any one option or increase or decrease intervention rates but rather act as an adjunct to care

The decision aid was designed for women to use at home or in the clinical setting, and takes about 30 minutes to complete. The aural component is available on both audiotapes and CDs so participants can choose which they prefer to use. After working through the decision aid, the woman brings her completed worksheet to her next antenatal appointment to discuss her provisional decision with her health care provider before arriving at her final decision. The worksheet is also useful for the practitioner, who can see rapidly from it what evidence the patient has considered, what her values and preferences on this topic are and which way she is leaning in her decision.

The decision aid was developed, pilot tested and revised with extensive consumer involvement, as outlined in the NHMRC guideline on preparing information for consumers[41]. Content was largely driven by consumers' questions and information needs as determined from the cross-sectional study[28] and from the process of drafting, pilot testing and re-drafting.

A number of draft decision aids (including workbook, audiotape/CD script, and worksheet), were developed and each subjected to pilot testing and revision as we obtained feedback. The process of testing and revising started with the project group. The next phase included a review by a group of national and international content experts, including decision aid experts, obstetricians, midwives, perinatal epidemiologists and psychologists. Once we were convinced that the content was accurate the decision aid was pilot-tested amongst consumers. There were several rounds of consumer review and refinement. We pilot-tested with members of consumer organisation (Maternity Alliance) and in a convenience sample of pregnant and recently pregnant women. The next draft was pilot-tested amongst pregnant women attending the antenatal clinic, who may or may not have had a breech presentation. And finally we formally pilot tested the decision aid with women who had a breech presentation in late pregnancy and were at the point of decision making. Pilot-testing results included: 95% of participants found the decision aid clear and easy to understand and 80% thought there was enough information for them to make a decision. Over 90% found it very helpful and nearly all women would recommend it to others. After reviewing the decision aid, women experienced a significant increase in their knowledge scores, less anxiety, had no difficulty making decisions and were satisfied with their decision.

This study aims to evaluate the ECV Decision Aid for women with a breech-presenting baby in late pregnancy. The decision aid is based on the most recently available evidence and will be evaluated to assess the impact on women's satisfaction with decision-making, knowledge, anxiety and pregnancy outcomes. If successful, the results could be applied to a improve consumer information and participation in clinical decisions across a wide spectrum of pregnancy care issues.

## Methods

### Specific aim

To compare the relative effectiveness of the decision aid with standard care in relation to women's knowledge, expectations, satisfaction with and participation in decision making, anxiety and decisional conflict. Secondary outcomes will include service utilisation and perinatal outcomes.

### Hypotheses

The primary hypotheses of the study are:

Use of the ECV decision aid by women with a singleton breech-presenting infant in late pregnancy

1. increases knowledge about breech presentation and the management options

2. reduces decisional conflict (uncertainty about the course of action)

3. increases satisfaction with decision making

4. reduces anxiety

The secondary hypotheses of the study are:

Use of the ECV decision aid by women with a singleton breech-presenting infant in late pregnancy does not influence

1. uptake of ECV

2. the proportion of women having a planned caesarean section for breech presentation at term

3. maternal and infant outcomes

### Study design

We will use a randomised trial with the following study groups to assess the impact of the decision aid:

Group 1: Usual care (usual antenatal care provider counselling on the management of breech presentation)

Group 2: Usual care + Decision aid with review by a research midwife

As randomisation will be done at the individual level, there is a risk of contamination of the usual care group if the usual care provider also reviews the decision aid with women in the study group. Therefore the decision aid will be reviewed with a research midwife and the usual antenatal care providers will be blinded to the exact content and format of the decision aid.

### Setting

Australian obstetric hospitals that offer external cephalic version.

### Participants/eligibility criteria

Women with a single breech-presenting baby at ≥ 34 weeks gestation and who are clinically eligible for ECV will be invited to participate in the trial.

*Exclusion criteria *are therefore those for ECV and include women presenting with a breech in labour, multiple pregnancies, previous CS, severe fetal anomaly, ruptured membranes and indications for CS anyway. The decision aid will be produced in English and will be designed to be simple and accessible for women with low levels of literacy. The use of audiotapes and graphics will further aid comprehension and ensure that, as far as possible, women with low English literacy need not be excluded.

### Procedures, recruitment, randomisation and collection of baseline data

**Figure 1:**
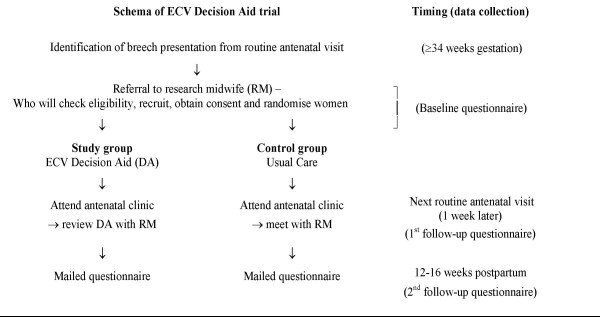
Schema of ECV decision aid trial

The study procedure will draw upon the usual schedule of antenatal visits becoming weekly in late pregnancy and the usual management of a breech-presentation in the late pregnancy (Figure 1). Women diagnosed with a breech-presenting baby at ≥ 34 weeks gestation will be asked to participate. The research midwife will explain the trial and obtain informed consent, collect baseline data and randomly allocate (using telephone randomisation) women to study or control groups. This is only a minor deviation from current practice. As women of child-bearing age are known to be very mobile, participants will be asked to provide alternate contact details (eg friend or relative) to enhance subsequent follow-up. Private obstetricians will be asked to offer their patients participation in the study. Those interested will be requested to come to the antenatal clinic for randomisation and recruitment. The private obstetrician will provide usual care.

### Intervention

The study group will receive the decision aid (workbook, tape or CD, and worksheet) and the control group will receive standard information on management options for breech presentation from their usual carer. The study group will be given the opportunity to work through the decision aid while in the antenatal clinic and/or to take home, which ever is most convenient. Many women will also want to discuss the decision with their partner. This pragmatic approach aims to assess the decision aid under the conditions most likely to be applied in real practice. At the next antenatal visit, women in the study group will review their decision aid worksheet and any questions with the research nurse.

### Follow-up 1

All women will be given a follow-up questionnaire to complete prior to their next antenatal clinic visit (see Outcome Measures below for more detail). All women choosing ECV will be given the opportunity to discuss the procedure with the obstetrician providing the service. This discussion is likely to include some of the probabilistic information in the decision aid but will occur after the follow-up questionnaire and will not influence the outcome measures.

### Follow-up 2

At 12–16 weeks post-partum all participants will be mailed a second follow-up questionnaire. This will assess women's satisfaction with their decision and the decision making process when the events are past and the outcomes known. (See Outcome Measures below for more detail). Questionnaires will be mailed with reply paid envelopes, with up to two reminder telephone prompts to non-responders.

### Blinding and contamination

As in many obstetric interventions double blinding is virtually impossible. The main outcomes of this study are self-reported and the women are clearly not blinded to their treatment allocation. However, we will institute a number of measures aimed at keeping antenatal staff blind to the treatment allocation and preventing contamination of the control group:

• Women will review the decision aid with the research midwife and complete the first questionnaire (primary outcome measures) prior to their next antenatal consultation

• Usual antenatal care providers will be blinded to the content and format of the decision aid

• Regular in-service (educational training) for the antenatal care providers to explain the trial protocol and to make clear the potential effect of unmasking or contamination.

• Monitoring decision aid distribution and keeping them locked up and only accessible by the research midwife

• Asking participants not to reveal their treatment allocation, or share their decision aid material with antenatal staff or other women. If participants do not want to keep their decision aid they will be asked to return it.

• Monitoring the "usual care" (control) arm by conducting a run-in period in which women found to have a breech presentation will be asked to complete the 1^st ^follow-up questionnaire. Thus we will have a baseline record of knowledge about ECV, anxiety and decisional conflict about the decision and satisfaction with the decision before the DA is in use. Comparison of the data obtained from this run-in period and the control arm will allow us to judge whether, and to what extent, contamination has occurred.

### Outcome measures

#### Baseline data collection

Brief baseline data will be collected to assess comparability of the study groups. The baseline assessment will include age, parity, brief socio-demographic data, highest level of education achieved, knowledge and anxiety as assessed by the state component of the short Spielberger anxiety scale[42].

#### Primary outcomes

The effectiveness of risk communication to aid patient decision making is best assessed by a combination of cognitive, affective and behavioural outcomes[43]. Thus the primary outcomes of the this study will be

• cognitive: change in knowledge and realistic expectations of the management options and possible benefits and risks of each option

• affective: anxiety, satisfaction with the decision, participation in decision-making and the amount of decisional conflict (uncertainty about which course of action to choose) experienced

• behavioural: actual decision taken and acted upon (see secondary outcomes)

Measures of knowledge and realistic expectations about options for the management of breech presentation and the benefits and risks of ECV will be specific to this project. Thus we will need to develop, and test these measures as part of the project.

Anxiety will be measured by the state component of the short Spielberger anxiety scale which has been extensively used and validated[42]. We do not anticipate the decision aid will increase women's anxiety but it is nevertheless important to document any increase or decrease in anxiety attributable to the decision aid.

Satisfaction with the decision will be assessed using the Satisfaction with Decision Scale. Satisfaction with Decision Scale (a very brief six item scale with high reliability) was developed specifically to assess satisfaction with health care decisions[44].

Participation in decision-making will be ascertained using the five-item Degner Control Preferences Scale[45]. This allows respondents to specify the degree of control in decision-making they wish to assume with their doctor.

Decisional conflict will be assessed by the Decisional Conflict Scale which has established reliability, good psychometric properties and is short (16 items)[46]. It has been used to evaluate a range of decision aids[35].

Because the decision about ECV must be made within a short timeframe, the outcomes will be measured as soon as practical after the consultation in which the ECV decision was made – prior to the next antenatal visit. For the primary outcomes this will be within one week of the decision being made (Figure 1, 1^st ^follow-up).

Satisfaction with the decision and anxiety will be measured again at 12–16 weeks postpartum as the last weeks of pregnancy and the week after birth are associated with a reduction in state anxiety[47]. We are interested to explore whether women's views of the decision making process, and the decision they ultimately made, may change with time to reflect on the experience (Figure 1, 2^nd ^follow-up).

#### Secondary outcomes

The aim of the decision aid is to assist patient decision making, and not to influence the direction of the decision taken. Nevertheless, we think it is important to collect service utilisation and pregnancy outcome data so we will record and compare the numbers of ECVs undergone and ECV success rate in both arms of the study, as well as recording and comparing rates of pregnancy complications and perinatal outcomes. Data on ECVs are already prospectively collected for quality assurance, these include fetal lie, parity, success rates and complications. Other perinatal outcomes will be obtained (with informed consent) from the existing computerised obstetric database. These outcomes include mode of delivery (vaginal, emergency or planned CS), enrolment to delivery interval, gestational age, birthweight, Apgar scores, perinatal deaths, Neonatal Intensive Care Unit admission, maternal haemorrhage (antepartum or postpartum) and length of stay.

### Statistical issues

#### Sample size

Sample size calculations for the trial (significance 0.05, power 0.8) were determined using the mean difference we would like to detect in women's decisional conflict scores and knowledge of options and outcomes. Compared with usual care, decisional conflict was shown in the most current systematic review to be significantly reduced by decision aids; the meta-analysed mean difference was -5.75, 95%CI -8.63, -2.87 (on a scale ranging from 1 lowest to 5 highest decisional conflict; median standard deviation 13.25)[48]. Assuming a mean difference of -5.75 and standard deviation 13.25, we would need approximately 84 women in each arm to demonstrate changes in decisional conflict. The meta-analysis also showed that for nine trials comparing decision aids and usual care, decision aids improved average knowledge scores by 18.75 points (out of 100) (95%CI 13.1 to 24.4, median standard deviation 20)[48]. To show such a difference, assuming mean difference of 18.75 and standard deviation of 20, would require only 18 women in each arm. Because we would like to be able to show differences in decisional conflict if they exist, we have used the larger sample size estimate. Although follow-up will be relatively short term, there will inevitably be some loss to follow-up. To allow for 10% loss to follow-up the sample size calculated above (84) is inflated by 10% to give the effective sample size of 92 women per arm and a total sample of 184 for the trial. This sample size is different from our original application for funding. Originally, we estimated a sample size of 310 women (155 in each arm). This was based on results from a 1999 systematic review of only 2 trials of decision aids versus usual care that had assessed decisional conflict[35]. Subsequent to the submission (January 2001) and funding of this protocol (March 2002) the systematic review was updated (2002) incorporating 6 trials that assessed decisional conflict[48]. At that time we revised the sample size estimate to incorporate the most current research evidence available. Ethics approval was obtained for the protocol amendment.

#### Data analysis

Analyses will be by intention to treat, including withdrawals and losses to follow-up. Study groups will be compared in terms of baseline characteristics. As this is a randomised trial, we would anticipate minimal differences in baseline characteristics. If however, important differences are found, these potential confounders will be adjusted for in the analysis of outcomes. For the primary outcomes, the mean score for each measure for each group will be compared using t-tests. If adjustment for confounders is needed a multiple linear regression model will be used. The secondary outcomes will be compared using chi-square tests of significance for categorical data and t-tests for continuous data. If adjustment for confounding is necessary logistic regression and multiple linear regression will be used respectively.

#### Interim analysis

An interim analysis will be conducted part way through the study and the results will be reviewed by an independent Data Monitoring Committee. Specifically the incidence of anxiety and decisional conflict in the two randomised groups will be determined after the first 150 women have been enrolled and data have been collected. If there is a significant increase in either of these outcomes at p < 0.01 (1-tailed) with the decision aid, the trial will be stopped. The trial will also be stopped of it is evident that no clear outcome will be obtained.

### Ethical considerations

We expect the project to provide ethical benefit. It is possible that some women may experience heightened anxiety as a result of receiving the decision aid during its evaluation by randomised trial. However, a systematic review of decision aids found they improved knowledge without increasing anxiety[33]. Nevertheless we will measure anxiety levels at baseline and follow-up to document any adverse effects. A trained research midwife will interview all women and obtain written consent for the trial. Women will be encouraged to discuss any concerns or anxiety about the project with the research midwife and/or with their usual antenatal care provider. Women will be reassured that they are able to drop out of the study at any time with no adverse effects on the management of their pregnancy. Participation will require women to complete brief self-report questionnaires during and after pregnancy. Working through the decision aid will take ~ 30 minutes and review of the decision and any outstanding questions will be at a routine antenatal visit.

The study has been approved by the Central Sydney Area Health Service Ethics Review Committee (Protocol no. X01-0067) and the University of Sydney Human Ethics Committee (Ref No. 3806).

### Confidentiality and data security

Participants in the trial will be identified by a study number only, with a master code sheet linking names with numbers being held securely and separately from the study data.

To ensure that all information is secure, data records will be kept in a secure location at the University of Sydney and accessible only to research staff. As soon as all follow-up is completed the data records will be de-identified. De-identified data will be used for the statistical analysis and all publications will include only aggregated data.

The electronic version of the data will be maintained on a computer protected by password. All hard copy patient identifiable data and electronic backup files will be kept in locked cabinets, which are held in a locked room accessed only by security code and limited staff.

Data files will be stored for 7 (seven) years after completion of the project as recommended by the NHMRC. Disposal of identifiable information will be done through the use of designated bags and/or a shredding machine.

### Outcomes and significance

This project will make an important contribution to a largely neglected aspect of pregnancy care, assisting informed participation by women in clinical decisions that affect their pregnancy. Involvement in decision making is a strong predictor of satisfaction with care in pregnancy and childbirth, yet there are only a few published decision aids for maternity care. A decision aid for the management of breech presentation is both timely and practical as there is new evidence supporting planned CS, dramatically altering the management options. Further, the randomised trial will provide high quality evidence about the effectiveness of the decision aid in supporting shared clinical decision making during pregnancy. If successful, the results of this project could be applied to improve consumer information and participation in clinical decisions across a wide spectrum of pregnancy care. Finally, if the decision aid increases the utilisation of ECV, in addition to reducing breech presentation and CS for breech presentation (and the associated increased hospitalisation and potential morbidities), some women may have more choice of where they give birth as breech presentation precludes birth in birth centres and small rural hospitals.

## Competing interests

The author(s) declare that they have no competing interests.

## Authors' contributions

CR, AB, CRG, BP, DHS were involved in the conception and design of the study. CR, NN and CRG were responsible for the drafting of the protocol and NN and CR were involved in the development and implementation of the study. All authors have read and given final approval of the final manuscript.

## Pre-publication history

The pre-publication history for this paper can be accessed here:


